# Environmental transfer parameters of strontium for soil to cow milk pathway for tropical monsoonal climatic region of the Indian subcontinent

**DOI:** 10.1038/s41598-022-11388-1

**Published:** 2022-05-09

**Authors:** P. Ujwal, I. Yashodhara, K. Sudeep Kumara, P. M. Ravi, N. Karunakara

**Affiliations:** 1 Centre for Advanced Research in Environmental Radioactivity (CARER), Mangalore University, Mangalagangothri, 574199 India; 2 Present Address: Department of Science and Humanities, Rajarambapu Institute of Technology, Sangli, Maharastra 415414 India

**Keywords:** Ecology, Environmental sciences

## Abstract

The radionuclide transfer between compartments is commonly described by transfer parameters representing the ratio of concentrations of an element in two compartments for equilibrium conditions. This is a comprehensive study on the soil-to-grass transfer factor (F_v_) and grass-to-cow milk transfer coefficient (F_m_) for stable strontium (Sr) for soil-grass (pasture)-cow (*Bos taurus)* milk environmental pathway under field conditions for a high rainfall tropical monsoonal climatic region of the Indian subcontinent. The study was conducted in the vicinity of the Kaiga nuclear power plant (NPP), situated ~ 58 km inland of the West Coast of the Indian subcontinent. A grass field was developed exclusively for this study, and two cows of the native breed were raised to graze on it. The soil, grass, and milk were analyzed to evaluate the F_v_ and the F_m_ values for the stable Sr. For comparison, several pasture lands and the cows raised by the villagers and a dairy farm were also studied. The F_v_ values were in the range 0.18—8.6, the geometric mean (GM) being 1.8. The correlations of F_v_ values with a range of physicochemical parameters are presented. The GM values for F_m_ were 2.2 × 10^–3^ d L^-1^ and 7.2 × 10^–3^ d L^-1^ for the two cows raised for this study, 2.6 × 10^–3^ d L^-1^ for those raised by the villagers, and 4.2 × 10^–3^ d L^-1^ for the dairy farm. The site-specific F_m_ value for the region was determined as 3.2 × 10^–3^ d L^-1^. The concentration ratio (CR), defined as the ratio of Sr concentration in milk to that in feed under equilibrium conditions, exhibited less variability (1.8 × 10^–2^—5.4 × 10^–2^) among the three categories of cows.

## Introduction

Naturally occurring strontium (Sr) has four stable isotopes: ^88^Sr, ^86^Sr, ^87^Sr, and ^84^Sr, with corresponding abundances of 82.5%, 9.8%, 7%, and 0.56%; the average concentration of this element in the earth's crust is 450 ppm^1–3^. Anthropogenic origin radioactive Sr (^90^Sr, T_1/2_ = 29.1 y) present in the environment is primarily due to the open-air detonation of nuclear weapons in the 1950s to 1960s, with an estimated ^90^Sr release of 6.3 × 10^17^ Bq to the atmosphere^4^. The 'Kyshtym Accident', in 1957 at the Mayak Production Association nuclear complex, released ~ 4.0 × 10^15^ Bq of ^90^Sr + ^90^Y into the atmosphere^5^. The accident at the nuclear power plant (NPP) in Chernobyl in 1986 released ~ 8.1 × 10^16^ Bq of ^89^Sr (T_1/2_ = 50.6 d) and 8.1 × 10^15^ Bq of ^90^Sr into the atmosphere^6^. The estimated release due to the Fukushima Daiichi NPP accident was ~ 2.0 × 10^15^ Bq of ^89^Sr and 1.4 × 10^14^ Bq of ^90^Sr^7^. Due to its short half-life, the global transport and the human exposure to ^89^Sr are not significant. On the other hand, due to the sufficiently long half-life, the nuclear weapons and nuclear accidents derived ^90^Sr were transported around the globe through the upper atmosphere and resulted in global fallout and contamination. Releases of ^90^Sr that occur during the routine operation of the NPPs are confined to the vicinity of the NPPs, and it is insignificant compared to the releases from weapons testing and accidents discussed just above.

The fate of the ^90^Sr in the environment is similar to the stable Sr. It enters into plants through foliar absorption of the atmospheric deposition and root uptake from soil. It bio-concentrates in the bones of both aquatic and terrestrial animals. Animals such as cattle, sheep, reindeer, etc., consume the contaminated plants and eventually transfer the radioactivity to humans through an ingestion pathway involving contaminated meat, milk or milk products. The degree of accumulation of Sr isotopes by plants depends on a range of physicochemical parameters and the concentration of competing element calcium in the soil and within the plant tissue. The uptake of Sr by plants depends on the soil calcium content, with higher uptake occurring in the soils with low calcium content^8^. In ruminants, the stable element status can affect the behaviour of a radionuclide analogue within the body; for example, the transfer of radiostrontium to milk declines as calcium intake increases.

Among different environmental transfer pathways which transfer radioactivity from the source of release to the population, the grass-cattle-milk-human pathway is important since anthropogenic radioisotopes of iodine (I), cesium (Cs), and Sr are transferred relatively quickly through it. For example, research has indicated that among agricultural products, cow (*Bos taurus)* milk and meat were the most significant contributors to ingestion dose in the Chernobyl-affected regions^9^. In addition, milk constitutes a significant component of the human diet; it is a staple food for infants and children. The radionuclide transfer between compartments is commonly described by transfer parameters representing the ratio of concentrations of an element in two compartments for equilibrium conditions. For example, the soil-to-grass transfer factor (F_v_) and grass-to-cattle milk transfer coefficient (F_m_) are defined by the following equations^10–13^:

1 $${\text{F}}_{{\text{v}}} = \frac{{{\text{Activity concentration in grass }}\left( {{\text{Bq kg}}^{{ - {1}}} ,{\text{ dry mass}}} \right)}}{{{\text{Activity concentration in soil }}\left( {{\text{Bq kg}}^{{ - {1}}} ,{\text{ dry mass}}} \right)}}$$

2 $${\text{F}}_{{\text{m}}} \left( {{\text{d L}}^{{ - {1}}} } \right) = \frac{{{\text{Activity concentration in milk }}\left( {{\text{Bq L}}^{{ - {1}}} ,{\text{ fresh mass}}} \right)}}{{{\text{Activity concentration in feed }}\left( {{\text{Bq kg}}^{{ - {1}}} ,{\text{ dry mass}}} \right) \, \times {\text{ Feed intake }}\left( {{\text{kg d}}^{{ - {1}}} ,{\text{ dry mass}}} \right)}}$$

For evaluating F_v_ and F_m_ for stable elements using the above equations, the concentration is expressed in mg kg^-1^ (dry mass) for the plant (or plant compartment) and the soil, whereas in mg L^-1^ (fresh mass) for milk.

For Cs, I, and Sr radioisotopes, several studies on F_v_ and F_m_ were published for temperate climates^1,14–29^. A very few studies on Cs and I radioisotopes have been reported for the Indian subcontinent as well^30–37^. The IAEA Technical Report Series (TRS) No. 472^10^ has listed 288, 104, and 154 studies on F_m_ for cow milk for Cs, I and Sr, respectively, with most of the data being related to the temperate environment.

The IAEA Modelling and Data for Radiological Impact Assessments (MODARIA II) programme (2016–2019) has led to the publication of IAEA TECDOC-1979^38^ listing revised F_v_ and F_m_ datasets for different elements. This document has collated data on F_v_  for radionuclides and stable elements in non-temperate environments (arid and tropical regions), and from a comparison of the data, it was concluded that crops grown in tropical environments have higher F_v_ values when compared to temperate and arid environments. Subsequently, a special issue of the Journal of Environmental Radioactivity (edited by Iurian^39^) was dedicated to supplementing the MODARIA II outcome. In this special issue, Doering^40^ has compiled data on F_v_ exclusively for the tropical regions from nearly 100 source references covering 36 elements, including data for radionuclides and stable isotopes for a variety of cereals, rice, vegetables, tubers, fruits, root crops, grass, tree leaves, herbs, etc. The geographical coverage included 21 countries of tropical regions spread across four continents (Africa, Asia, Australia, and South America). Similarly, Rout^41,42^ has reviewed F_v_ data published for terrestrial plants and rice of the Indian subcontinent. It is imperative to note from these reviews that in comparison to a large number of publications for the temperate regions, only 10 publications have reported F_v_ data for ^90^Sr or stable Sr for food crops and plants^30,43–51^, and only one publication^45^ reported data for grass for the tropical environment.

Similarly, MODARIA II has also led to the publication of the IAEA TECDOC-1950^52^, effecting substantial revision to F_m_ datasets for cow milk published previously by the IAEA^10,11^. From 118 data points, a mean value of 1.5 × 10^–3^ d kg^-1^ (range: 1.5 × 10^–5^—4.3 × 10^–3^) was arrived at for the Sr F_m_ for cow milk, with most of the data coming from temperate regions. Earlier, Howard^12^ published revised Cs, I, and Sr F_m_ datasets for cow milk from the MODARIA II and have listed (i) the publications which were considered in IAEA TRS 472^10^ and (ii) those added or removed from it for establishing the revised datasets subsequently given in the IAEA TECDOC-1950^52^. Tagami^13^ reported a comprehensive review on the soil-to-animal transfer factors for the tropical climatic regions and opinioned that information available on radioecology of tropical plant and animal species is very little when compared to those of temperate regions. These authors have also commented that much of the soil-to-animal databases reviewed and listed in their publication for the tropical environment are related to Australian data^53^, which came from “grey literature sources not generally available to the broader scientific community”^13^. Our observation is that the above-referred review publications and the IAEA documents do not list F_m_ data for Sr for cow milk for tropical regions.

Therefore, generating experimental databases through site-specific studies in the vicinity of nuclear facilities in the tropical climatic region is essential for accurate dose assessments. Maria and Florou^54^ have concluded that climatic types may be linked to the transfer of radionuclides from soil to grass. Tropical soils are more strongly weathered, have less nutrients, and are often significantly different from temperate regions; hence, plant uptake characteristics in such soils are different. In addition, higher rainfall and temperature in tropical regions lead to faster leaching of elements from the soil profile resulting in reduced bioaccumulation due to poor bio-accessibility^13^. Also, the F_m_ is influenced by the dry matter intake (DMI) of the animal, the milk yield, body mass, breed type, etc.^12,23,55^. Large differences in these parameters have been reported for tropical and temperate regions; for example, the dairy farm cows of temperate regions have high milk yield (generally ~ 30 L d^-1^) due to improvement in animal breeding^12^, and DMI is up to 40 kg d^-1^^56^ with a guidance value of 16 kg d^-1^^10,57^. The milk yield and DMI depend on the live weight; for a cow with a 770 kg live weight and milk yield of 30 L d^-1^, the DMI was estimated to be 24.5 kg d^-1^^12^. On the other hand, the live weight of the native breed cows in villages in the tropical region (such as the Indian subcontinent) is significantly less and often yields < 3 L d^-1^ milk and is supplemented with negligible nutrient feed^35,58^. Establishing a database for such animals is also essential because, as demonstrated in our previous publication, the F_m_ for ^137^Cs was an order of magnitude greater for these animals when compared to high milk-yielding dairy farm animals^35^.

With the objective of establishing a site-specific database on F_v_ and F_m_ for different radionuclides and stable elements for a tropical high rainfall region for accurate radiation dose assessment, a detailed study was initiated by the Centre for Advanced Research in Environmental Radioactivity (CARER) in the year 2010 in the vicinity of pressurized heavy water (PHWR) NPP located at ~ 58 km east of the West Coast of India. This NPP site is characterised by unique topographical and monsoonal climatic conditions, as explained in  the following section. The findings of the study on I and Cs F_v_ and F_m_ values for this NPP site were reported in our previous publications^34–37,58,59^, and the data on Cs F_v_ and F_m_ have been listed in the IAEA documents^38,52^. In addition, an extensive database on soil-to-rice plant F_v_ for naturally occurring radionuclides and ^137^Cs for this NPP site were published^36^. Now, we report the determination of site-specific data for F_v_ and F_m_ for stable Sr for the soil-to-grass-to-cow milk pathway for equilibrium situations.

## Materials and methods

### Study region

**Figure 1:**
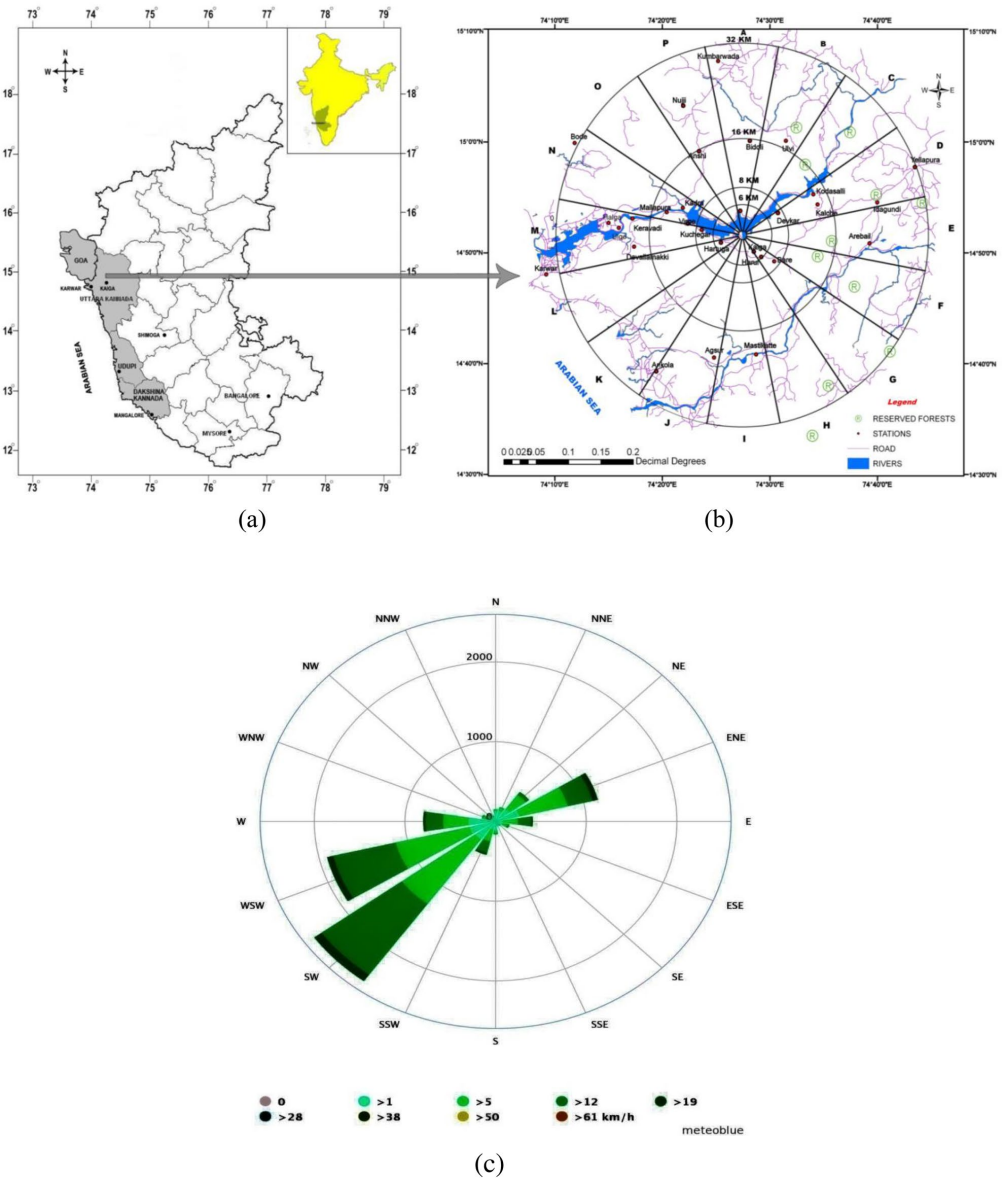
(**a**) Map of the West Coast region of India, (**b**) Kaiga region and the sampling stations, and (**c**) the wind rose for the Kaiga region. The map was drawn using QGIS software (version QGIS-OSGeo4W-1.5.0–13,926, link: QGIS-OSGeo4W-1.5.0–13,926-Setup.exe).

Kaiga (14° 52′18.5″N, 74°24′15.8″E) is situated on the foot of the world-famous Western Ghats and ~ 58 km east (aerial distance is ~32 km) of the coastal Karnataka town of Karwar, where four pressurized heavy water reactors (PHWR) each of 220MWe are in operation. The first two reactors (units 1&2) started power production in 2000, and the third and fourth reactors (units 3&4) in 2007 and 2011, respectively. This NPP is on the valley of the Kali River, which originates in the Western Ghats^60^. Due to the dense forest and hills with altitudes of 40  to 600 m, this region is beset with unique meteorological and ecological attributes^3,35,36,60–62^. The dominant wind direction is south-westerly and westerly during the south-west monsoon and summer seasons and north-easterly during winter. The tropical monsoon brings an annual rainfall between 4000—5000 mm y^-1^. Relative humidity at Kaiga varies from 17.7 to 99.9%, and ambient temperature varies from 13.3 to 41.0℃^30^. The soil is of lateritic origin and dark brown^3,35,62^. The population density in the villages is very low. Figure 1a–c, respectively, present the map of the Kaiga region, an enlarged view of the 32 km radius region of the NPP, and the wind rose diagram.

### Grass fields for the study

A grass field of size ~ 2800 sq. m was developed as an experimental field at an aerial distance of ~ 6 km (Kuchegar, 14º52′2.5" N, 74º22′41.1" E) from the NPP (Fig. 1b). This field was open land, and *Pennisetum purpureum* (Schum.) (known as napier grass), used as forage on the dairy farm, was planted and allowed to grow naturally^3,59^. For a comparative study, nine pasture lands in the neighbouring villages and a grass field maintained by a dairy farm at ~ 12 km (Mallapura, 14˚54ʹ05.6ʺ N, 74˚19ʹ56.9ʺ E) from the NPP were chosen. The predominant grass species in the pasture lands in the villages was *Ischaemum indicum* (Houtt.). The grass in all the fields was grown without applying any external fertilizers.

### Cows for collecting milk samples

Three categories of cows were identified for collecting milk samples. They are (i) the two cows raised for this study (from now on, referred to as cow 1 and cow 2), (ii) cows raised by the villagers and grazed in pasture lands, and (iii) cows of the dairy farm. The cows raised exclusively for this study were "Malnad gidda", a breed native to the region. The typical attributes of this breed are (i) small to medium in size, (ii) good resistance against pests and diseases, (iii) yield less milk (maximum yield ~ 4 L d^-1^, but often yield < 1.5 L d^-1^), and (iv) graze in hilly regions. Generally, these cattle are fed with minimal supplement feed; their dietary requirements are met through grazing in the pasture lands^35,58^.

Cow 1 and cow 2 were maintained with a similar feeding pattern as that followed by the villagers. The body mass of cow 1 was 400 kg, and cow 2 was 225 kg. They grazed in the experimental grass field. Raising the two cows exclusively for this study allowed the determination of the site-specific database on DMI (kg d^-1^) and the milk yield (L d^-1^). The milk yield of these animals was ~ 2 L d^-1^ in the early lactation period and < 1 L d^-1^ in the later stages, with cow 2 yielding significantly less milk when compared to cow 1. To ensure the availability of an adequate volume of milk for the analysis, cow 1 and cow 2 were fed with 1 kg d^-1^ of supplement feed (groundnut silage). The dairy farm cattle were high milk yielding Holstein Friesian breed, with a typical yield of 12–15 L d^-1^. To achieve a commercially viable milk yield, these cattle are fed with a substantial quantity of nutritious supplement feed^35^.

### Sample collection and preparation for measurements

Soil was sampled at 0–10 cm horizon in the grass fields since this depth represents the root zone^57,63^. The green leafy portion of the grass above a few cm from the surface of the soil was sampled^35^. Within a grass field, 8–10 subsamples of these matrices were collected at different points. A composite sample of each of these matrices (~ 2 kg each) was obtained by thoroughly mixing several subsamples collected within a grass field. Samples of milk from the three categories of cows were collected periodically. The sampling campaign was for two years duration. The samples of the supplement feed were also collected for analysis. A total of 95 soil samples, 95 grass, 4 supplementary feed, and 59 milk samples were collected for this study.

The procedure followed for processing the samples was discussed in an earlier publication^35^, and they are prescribed in EML^64^ and the IAEA^63^ documents. In the case of soil, a subsample was set aside to determine physicochemical parameters. The remaining portion was dried at a constant temperature of 105 °C for 24 h to remove water content completely. The grass and milk samples were dried at 105 °C, charred over a low flame, and ashed at 450 °C in a muffle furnace. Finally, the accurately weighed mass (0.5–0.7 g) of a processed sample was subjected to chemical digestion in a microwave-based sample digestion system (ETHOS™, closed vessel type, Milestone, Italy) to get a clear solution and taken for the determination of Sr concentration^35,65^.

The physicochemical parameters determined for the soil samples and methods employed were: (i) pH by 1:2 soil–water system and using a glass electrode pH meter, (ii) conductivity by 1:2 soil–water system and a using platinum dip type conductivity cell, (iii) organic matter content by loss of mass due to ignition at 450 °C, (iv) cation exchange capacity (CEC) by sodium exchange capacity method, and (v) particle sizes by hydrometer method^66^.

### Determination of Sr concentration

Previous studies have shown that activity concentration of ^90^Sr in the soil of most of the locations of Kaiga and West Coast region was below detection level^30,67,68^. These authors have analyzed the samples by radiochemical separation and counting the activity concentration in a β-counting system having a minimum detection level of < 1 Bq kg^-1^^69^. ^90^Sr activity concentration reported for the Kaiga region by Siddappa^68^ had a maximum value of 0.72 Bq kg^-1^. Similar values were reported by Joshy^30^, with values in the range < 1.1—2.2 Bq kg^-1^ for soil and < 1.2 – 4.5 Bq kg^-1^ for leaves of wild plants of the Kaiga region. A recent report of a long-term environmental assessment programme in the public domain of the Kaiga NPP has shown that the activity of ^90^Sr in soil, sediment, grass and leaf samples was < 0.5 Bq kg^-1^^67^.

In the initial stages of the present study, 12 milk samples were analyzed for the ^90^Sr following the method described in IAEA^63^, which involved the radiochemical separation of ^90^Sr, allowing for the ingrowth of ^90^Y, and counting the activity in a low background counting system. The activity in all these samples was < 0.1 Bq kg^-1^. Therefore, we have used stable Sr to determine the F_v_ and F_m_. Since the radioisotopes and their counterparts follow the same pathway, it can be used to estimate the transfer of ^90^Sr in food chains^70^. Furthermore, stable isotopes were widely used as analogues to estimate the transfer of radionuclides because of diminishing inventories of some of the radionuclides^40^. The concentration of stable Sr in µg kg^-1^ levels could be measured by analytical techniques such as atomic absorption spectrometry (AAS) or inductively coupled plasma mass spectrometry (ICP-MS). 

In this study, the Sr concentration in the digested samples was determined using an AAS (GF-300Plus, graphite furnace with PAL-3000 automatic sampler, GBC, Australia), which offers a minimum detection level (MDL) of 0.004 μg mL^-1^ at a 95% confidence level. The instrument was calibrated using Sr standards (MERCK, Germany), and the IAEA reference materials (IAEA Soil 7, IAEA-153 milk powder) were used for quality assurance in the measurements. Type-I ultrapure water, conforming to the American Society for Testing and Materials (ASTM), was used for sample processing. The Ca concentration in the digested soil samples was determined by AAS (with flame oxidizer) in the same way^71^.

### Calculation of F_v_ and F_m_ values

The F_v_ value was evaluated from the Sr concentrations in soil and grass using the following relation^10,35^:

3 $${\text{F}}_{{\text{v}}} = \frac{{{\text{C}}_{{\text{g}}} }}{{{\text{C}}_{{\text{s}}} }}$$

where, C_g_ and C_s_ are the Sr concentrations (mg kg^-1^, dry mass) in grass and soil, respectively.

The F_m_ (d L^-1^) value was calculated from the following relation^10,35^:

4 $${\text{F}}_{{\text{m}}} = \frac{{{\text{C}}_{{\text{m}}} }}{{{\text{C}}_{{\text{f}}} \times {\text{I}}_{{\text{f}}} }}$$

where, C_m_ is the Sr concentration in the milk (mg L^-1^, fresh mass), C_f_ is the Sr concentration in the feed (mg kg^-1^, dry mass), and I_f_ is the DMI (kg d^-1^, dry mass). The denominator in Eq. (4) is the ingestion rate of the element (mg d^-1^), and it was arrived at by considering the concentrations of Sr in grass and supplement feed.

### Determination of DMI

Establishing the F_m_ value necessitates using the site-specific data on DMI by the cattle. This information was obtained through a demography survey conducted during 2008 – 2013 in the vicinity of the NPP^35,58^. In this programme, 186 cows from 106 households of 12 villages were surveyed in the vicinity of the NPP to generate a database on the intake of water, DMI (supplement feed, grass and forage) and milk yield through feedback obtained from the questionnaires and in-situ measurements^35,58^. Although this survey was performed during 2008–2013, the database on fodder regime is expected to be valid even now due to minimal change in the animal rearing practices in the region.

In addition to this, measurements based on stall-feeding were conducted to determine the grass intake by the cattle^35,58^. In these experiments, cows 1 and cow 2 were confined to the stalls for 24 h, and a known quantity of fresh grass was offered. The mass of the unconsumed portion at the end of 24 h was determined. In addition to experimental determination, the daily intake of dry matter was also calculated theoretically, based on the following expression^35,72^:

5 $${\text{DMI }} = \frac{{{\text{BWT }} \times {\text{ PBWT}}}}{100}$$

where,

DMI is the daily dry matter intake (kg d^-1^).

BWT is the bodyweight of the cow (kg).

PBWT is the percentage of cow's body weight to be fed per day (%).

For cow 1, BWT was ~ 400 kg, and the corresponding value of PBWT as given in NRC ^72^ is 2.2%. For Cow 2, these values were ~ 225 kg and 2.2%, respectively.

## Results and discussion

### Physicochemical properties of soil

As stated previously (section "Introduction"), the extent of uptake of Sr isotopes by plants through roots is strongly affected by a range of physicochemical parameters of the soil and the concentration of the essential nutrient element Ca. In Table 1, the results of some of the physicochemical parameters of the soils, which are relevant for explaining the soil to grass transfer of Sr, are listed. The important observations were (i) the soil is acidic (mean value of pH was ~ 5), (ii) due to low clay content (particle sizes < 2 µm) and a higher percentage of particle sizes of > 2 µm, it belongs to the sand group; when categorized as described in IAEA-TRS-472 ^10^, (iii) have lower Ca content, an essential plant nutrient element and analogues of Sr, (iv) low CEC, and (v) low organic matter content. These soil parameters are generally responsible for the variation of F_v_ values, both directly and indirectly; hence, knowledge of these parameters is essential to predict the extent of soil-to-grass transfer of radionuclides.

**Table 1:** Physicochemical parameters of the soils.

Grass field	Soil parameters								
	pH	Conductivity (μS cm^-1^)	OM content (%)	Cation exchange (meq/100 g)	Particle size fraction			Ca (mg kg^-1^)	Soil group^c^
					< 2 μm (%)	2 – 50 μm (%)	> 50 μm (%)		
Experimental grass field ^[36^^]a^	4.2–6.9 (5.1)^b^	46.9–554 (318)	3.4–8.2 (5.6)	1.7–15.8 (6.3)	0–7.0 (3.1)	62.0–74.0 (70)	19.0–37.0 (28)	2.6–209 32.4	Sand ^10^
Pasture lands [50]	4.1–6.5 (5.0)	88–663 (467)	3.8–13.8 (8.1)	1.3–24.5 (7.3)	0–13.0 (4.8)	46.0–79.0 (63)	11.0–54.0 (33)	1.0–184 40.7	Sand^10^
Dairy farm [9]	4.8–6.0 (5.2)	103–677 (310)	3.2–4.6 (3.9)	2.5–2.6 (2.5)	0–7.0 (3.6)	52.0–78.0 (62)	20.0–48.0 (37)	13.7–34.2 20.8	Sand^10^

^a^Values within square brackets in column 1 represents samples analyzed.

^b^Values within parenthesis represent the arithmetic mean corresponding to the respective range.

^c^Soil group is based on the criteria described in IAEA-TRS-472^10^.

Soil pH affects Sr uptake in tropical systems; the bioavailability is generally higher at lower pH values because acidic soils favour the dissolution of Sr isotopes and Ca in the soil. Soil with reduced CEC and lower pH, clay and dissolved concentration of stable nutrient analogues, typical of strongly weathered tropical high rainfall regions such as the pasture lands of Kaiga, may lead to greater bioaccumulation of Sr isotopes^13^. On the other hand, in soils of tropical farms and forests with high organic matter, CEC and Ca content can reduce the bioaccumulation of Sr because of the greater strength of sorption of this element in such soils and preferential uptake of Ca. But, because of the reduced CEC, organic matter and Ca contents, we can infer that for the pasture land soils of the Kaiga region, the F_v_ value of Sr is expected to be higher.

### Concentration of Sr in soil

Table 2 (column 3) presents the concentration of stable Sr in the soil of grass fields, and it ranged from 0.14 to 64.3 mg kg^-1^ (GM = 10.2 mg kg^-1^). The ANOVA test confirmed at a 95% confidence level that the concentrations of this element in the soil are not significantly different across the experimental field, pasture lands and dairy farm fields. Previously reported values^70,73^ for the Kaiga region are in the range of 0.3—27.3 mg kg^-1^, which is similar to the values recorded in our study. The regression analyses revealed that (i) the correlation coefficient (R = 0.246, p > 0.05) between soil OM and Sr concentration was not statistically significant, and (ii) higher concentration values of this element in soil occur at low soil pH, as evidenced by the statistically significant correlation coefficient; R = -0.501, p < 0.05.

**Table 2:** Concentration of Sr in soil and grass and corresponding F_v_ values.

Details of the grass fields	Parameter	Sr concentration (mg kg^-1^, dry mass)		F_v_
		Soil	Grass	
Experimental fiel d[36]^a^	Range	3.2–64.3	10.9–88.0	0.31–8.6
	Mean	17.0	25.6	2.3
	SD^b^	13.7	16.3	1.9
	GM	12.6	22.4	1.7
	GSD^c^	2.3	1.6	2.4
	Median	14.4	20.2	1.8
Pasture lands [50]	Range	0.14–53.1	2.7–59.4	0.18–8.4
	Mean	12.3	20.0	2.5
	SD	12.3	14.6	2.1
	GM	7.9	14.7	1.8
	GSD	3.0	2.2	2.2
	Median	7.7	15.1	1.5
Dairy farm [9]	Range	0.14—4.3	5.0–11.0	1.1–3.5
	Mean	2.4	8.7	2.3
	SD	2.1	3.2	1.7
	GM	1.2	8.2	2.0
	GSD	2.4	2.6	2.1
	Median	2.8	10	2.3

^a^Values within square brackets in column 1 represents the number of samples analyzed (soil and grass each).

^b^SD denotes standard deviation.

^c^GSD represents the geometric standard deviation (unitless).

### Sr concentrations in grass

The concentration of stable Sr in the grass of the experimental field and pasture lands ranged from 2.7 to 88.0 mg kg^-1^ (GM = 15.1 mg kg^-1^) (Table 2, column 4). The previously reported range for leaves of different plants species of Kaiga was 3.2–50.4 mg kg^-1^^70,73^. ANOVA test proved that Sr concentration in grass grown in the experiment field was similar to those recorded for pasture lands (at 95% confidence level, the mean values of two data sets were not significantly different, F_obs_ = 0.73, F_0.05_ [1, 89] = 3.94).

**Figure 2:**
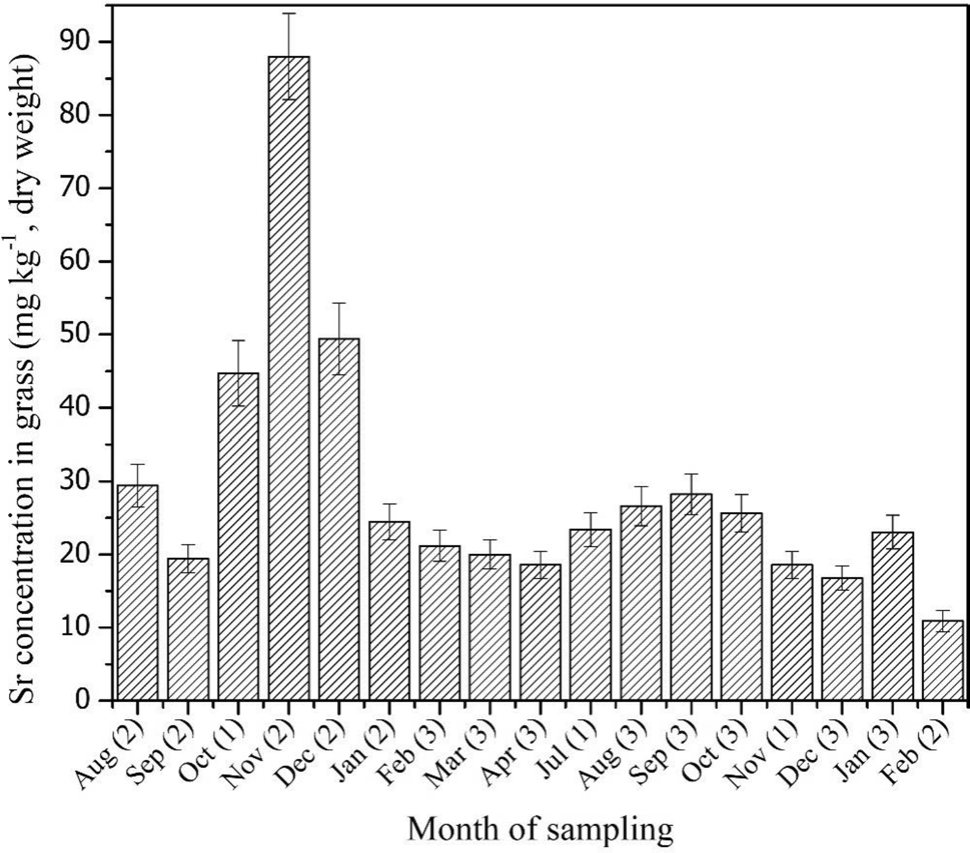
Monthly variation of stable Sr concentration in the grass (experimental field). The number of samples analyzed for each month is given in the parenthesis.

Figure 2 presents the monthly variation of Sr in the grass of the experimental field. Except during the period October-December the concentration remained reasonably uniform. Similar observations were recorded in pasture lands and the dairy farm field. The grass was manually defoliated regularly to feed the cattle in the shed on the dairy farm. On the other hand, the animals defoliated the pasture lands during grazing in the other two categories of fields. According to Ehlken and Kirchner ^74^, pasture cropped repeatedly develops a shallow root system leading to enhanced uptake of elements and nutrients from top soil, which may suppress seasonal variations in the concentrations of the above-ground parts. However, it is to be noted that seasonal variations in the uptake would have very little importance in the event of a release from a nuclear facility since direct deposition from the atmosphere is a much more efficient contamination mechanism for anthropogenic radioisotopes of Cs, Sr and I.

### F_v_ values for stable Sr

The F_v_ values of Sr ranged between 0.18–8.6, with a GM value of 1.80 (Table 2, column 5), and they were similar for the experimental field and other pasture lands even though the grass species in these fields differed. The ANOVA test confirmed that the mean values were not significantly different at 95% confidence level, with F_obs_ = 0.024, F_0.05_ [1,88]= 3.949.

Table 3 compares the Sr F_v_ values recorded in this study with those reported by other investigators. The published data compiled in the table include both stable and radioactive Sr. The mean value derived from this study is the same as that reported previously for the Kaiga region ^10,38,70^. Data compilation for temperate and sub-tropical regions of the world had ranges of 0.056—7.3 and 0.069–0.91, respectively, with corresponding mean values of 1.3 and 0.78. The only publication listed for the tropical region in the IAEA document^38^ is for Bangladesh^45^, and the values range from 0.79 to 0.91, with a mean of 0.84. Depending upon the soil properties, plants species and climatic conditions, the F_v_ values listed in IAEA^38^ vary up to four orders of magnitude. According to IUR^75^, F_v_ for this element is greater than unity. Comparing the F_v_ values obtained from field studies at Tianwan NPP, China, for different types of vegetables and grass with the worldwide data listed in the IAEA^11^, Lu^76^ have observed that their values were lower by 1.5–2.8 times and have remarked that most of the IAEA data were derived from pot/lysimeter experiments using radionuclides or experiments in which equilibrium conditions were not achieved. In this context, the present study has significance because it was performed for field conditions of a tropical monsoonal climatic region for which not many publications on F_v_ values for grass are reported, as discussed in section "Introduction".

**Table 3:** Comparison of the Sr F_v_ values for grass.

Region	Range	Mean	Reference
Kaiga, India	0.18–8.6	2.4 (1.8)^b^	Present study
Kaiga, India	^a^	2.4	^70^
Kaiga, India	^a^	< 0.80 ^c^	^89^
World-wide (Temperate environments)	0.056–7.3	1.3	^10^
Bangladesh (Tropical environment)	0.79–0.91	0.84 (0.84)	^38,45^
World-wide (Sub-tropical environments)	0.69–0.91	0.78	^10^
World-wide (Arid environments)	0.02–1.7	0.72 (0.16)	^38^

^a^Data not available.

^b^Value within the parenthesis represents GM.

^c^Denotes the F_v_ values for ^90^Sr.

The association between the soil parameters and F_v_ has been studied to identify those parameters which influence Sr uptake by the grass. As explained previously, the uptake of Sr is higher in plants grown in the soil with low OM content^8,13^. This is because of the fixation of Sr to the humic substance of the soil^77^. The linear correlation analysis for the data obtained in this study indicated a weak correlation between the two parameters (R = − 0.159, p > 0.05).

The F_v_ values were weakly correlated with soil clay content (R = -0.13, p > 0.05), soil moisture content (R = -0.092, p > 0.05) and the CEC (R = 0.173, p > 0.05). It must be emphasised here that the physicochemical parameters varied in a narrow range since the study was confined to a small region in the vicinity of a specific NPP site. To delineate the influence of the soil parameters on the transfer of elements to plants, one needs to conduct studies in different types of soils with widely varying physicochemical properties. The Sr F_v_ values were evaluated against the Ca concentration. Although the statistical analysis between the Sr F_v_ values and soil Ca concentration indicated that, in general, the Sr F_v_ increased with the decreasing Ca content in the soil, as evidenced by the negative sign on the correlation coefficient (R = − 0.37, p > 0.05). However, it was not statistically significant.

### Sr concentration in milk

**Figure 3:**
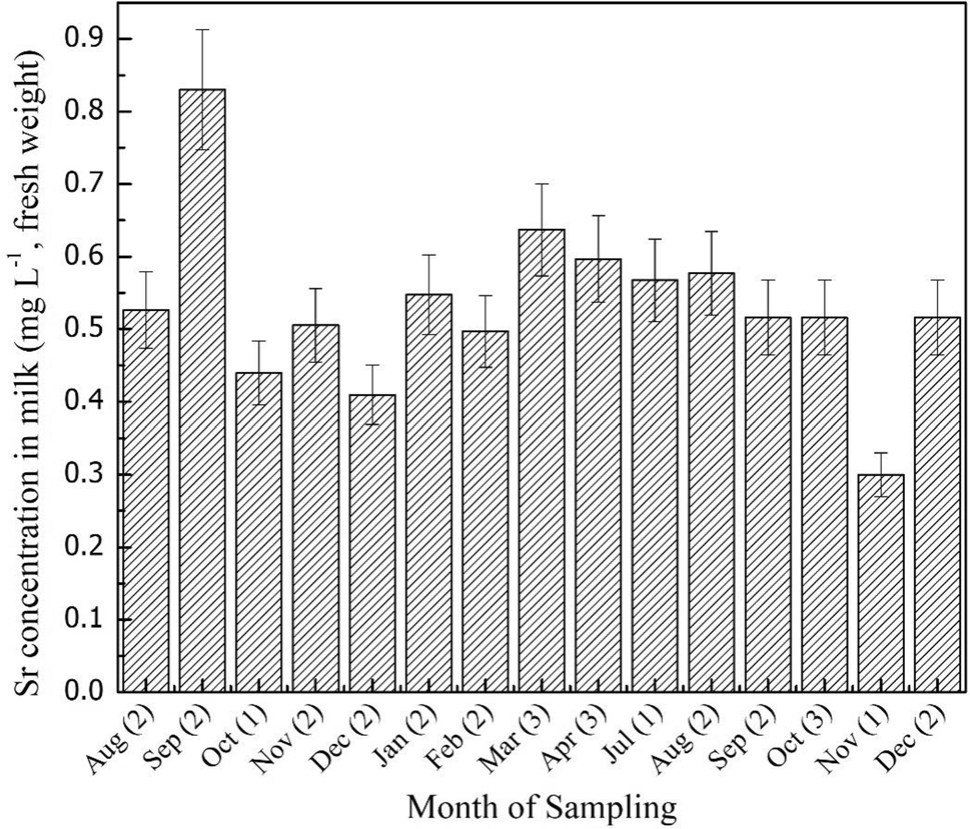
Monthly variation of stable Sr concentration in milk.

Table 4 presents the stable Sr concentration in milk of the cows raised for this study and the corresponding F_m_ values. The corresponding datasets for cows raised by the villagers and the dairy farm are presented in Table 5. For cow 1 and cow 2, the Sr concentration in the milk had GM values of 0.52 mg L^-1^ and 0.50 mg L^-1^, respectively. These are similar to that recorded for the cows raised by the villagers and the dairy farm (GM values for both categories were the same, 0.45 mg L^-1^). The statistical test (ANOVA) confirmed that the mean values of concentration of the three groups of animals were not significantly different (for example, F_obs_ = 0.506, F_0.05_ [1.48]= 4.043 for the datasets of cow 1 and cow 2 and those raised by the villages). The Sr concentration in the milk was monitored for both cow 1 and cow 2 throughout the year, and it remained uniform, as shown in Fig. 3.

**Table 4:** Stable Sr concentration in grass grown in the experimental field and milk and F_m_ values for the cows raised for the study.

Element	Parameter	Cow 1 (DMI = 8.3 kg d^-1^ grass + 1 kg d^-1^ supplement feed)			Cow 2 (DMI = 3.7 kg d^-1^ grass + 1 kg d^-1^ supplement feed)		
		Concentration in grass* (mg kg^-1^, dry mass) [17]^a^	Concentration in milk* (mg L^-1^, fresh mass) [17]	F_m_ (d L^-1^)	Concentration in grass* (mg kg^-1^, dry mass) [19]	Concentration in milk* (mg L^-1^, fresh mass) [13]	F_m_ (d L^-1^)
Sr	Range	17.4–88.0	0.33–0.83	7.5 × 10^–4^-5.4 × 10^–3^	11.3–52.1	0.3–0.69	2.2 × 10^–3^-1.4 × 10^–2^
	Mean	31.1	0.53	2.5 × 10^–3^	20.9	0.51	8.1 × 10^–3^
	SD^b^	15.5	0.13	1.3 × 10^–3^	11.0	0.10	3.9 × 10^–3^
	GM	28.2	0.52	2.2 × 10^–3^	18.9	0.50	7.2 × 10^–3^
	GSD^c^	1.5	1.3	1.90	1.4	1.3	1.7
	Median	27.6	0.54	2.7 × 10^–3^	18.1	0.52	6.7 × 10^–3^

^a^Values within the square bracket represents samples analyzed.

^b^SD represents standard deviation.

^c^GSD represents the geometric standard deviation (unitless).

^d^Sr concentration in the supplement feed was below the detection limit.

**Table 5:** Stable Sr concentration in pasture land grass and milk of cows raised by the villagers and corresponding F_m_ values.

Grass fields	Parameter	Concentration in grass (mg kg^-1^, dry mass)	Concentration in milk (mg L^-1^, fresh mass)	F_m_ (d L^-1^) (DMI = 8.3 kg d^-1^ for cows raised by the villagers, 13 kg d^-1^ for dairy farm)^d,e^
Pasture lands [grass = 50 milk = 20]^a^	Range	5.0–43.5	0.11–1.0	4.8 × 10^–4^-9.1 × 10^–3^
	Mean	22.5	0.49	3.3 × 10^–3^
	SD^b^	11.8	0.18	2.3 × 10^–3^
	GM	18.9	0.45	2.6 × 10^–3^
	GSD^c^	2.2	1.6	2.0
	Median	23.3	0.48	2.5 × 10^–3^
Dairy farm [grass = 9 milk = 9]	Range	5.0–11.0	0.27–0.73	1.8 × 10^–3^ – 1.1 × 10^–2^
	Mean	8.7	0.49	5.5 × 10^–3^
	SD	3.2	0.23	4.9 × 10^–3^
	GM	8.2	0.45	4.2 × 10^–3^
	GSD	1.9	1.6	2.5
	Median	10.0	0.46	3.5 × 10^–3^

^a^Values within the square bracket represents samples analyzed.

^b^SD represents standard deviation.

^c^GSD represents the geometric standard deviation (unitless).

^d^DMI by cows raised by the villagers is 8.3 kg d^-1^ of grass (no supplement diet); for the dairy farm, it is 4.4 kg d^-1^ of grass + 8.6 kg d^-1^ of nutrient supplement diet.

^e^Sr concentration in the supplement feed was below the detection limit.

Anderson^78^ reported a value of 0.42 mg L^-1^ for stable Sr in cow milk, and a value of 0.17 mg L^-1^ was reported by Hurleyw^79^. As outlined in section "Determination of Sr concentration", the milk analyzed for ^90^Sr in the present study showed activity < 0.1 Bq L^-1^. Values reported for ^90^Sr by previous investigators were 0.05 Bq L^-1^ for Mangalore, South India^69^, 0.11—0.16 Bq L^-1^ for Mumbai, India^80^. Those reported for other countries ranged from 0.05 to 3.1 Bq L^-1^^15,21,81–83^.

### F_m_ values

The F_m_ values for stable Sr are listed in Table 4 for the two cows raised for this study. Table 5 present the values for the other two categories of animals. The site-specific data on DMI (8.3 kg d^-1^ and 3.7 kg d^-1^ grass for cow 1 and cow 2, respectively, and 1 kg d^-1^ supplement feed), measured through stall-feeding (section "Determination of DMI" ), were used for F_m_ calculation. The considerable difference in DMI between cow 1 and cow 2 can be related to the significant disparity in their body mass: cow 1 was 400 kg, and cow 2 was 225 kg ^12,35^.

Stall-feeding experiments were not performed with the cows raised by villagers. But, several measurements of the grass intake during the captive period, i.e., after cattle return to the stalls , were performed. For the captive period (18:00 h to 6:00 h), DMI was ~ 3.3 kg d^-1^ (mean value); this value is similar to the DMI of cow 1 for the captive period. Moreover, the body mass of the cows raised by the villagers was similar to that of cow 1. Hence, the DMI of cow 1 (8.3 kg d^-1^ grass, dry mass) was used as the representative value for this group. Also, the data obtained from the demographic survey supported the above viewpoint. In the case of dairy farm cattle, the DMI was measured, and the mean value was 13 kg d^-1^ (8.6 kg of supplement feed + 4.4 kg of grass, dry mass). Analysis of samples of supplement feed showed that the stable Sr concentration was below the detection limit.

The GM values of F_m_ were 2.2 × 10^–3^ d L^-1^ for cow 1 and 7.2 × 10^–3^ d L^-1^ for cow 2 (Table 4, columns 5 and 8), 2.6 × 10^–3^ d L^-1^ for the cattle raised by the villagers (Table 5, column 5), and 4.2 × 10^–3^ d L^-1^ for the dairy farm cattle (Table 5, column 5). Statistical analysis (ANOVA test) of the F_m_ datasets for cow 1 and cow 2 and those raised by the villagers confirmed that the mean values of the two datasets are not different at a 95% confidence level (F_obs_ = 3.028, F_0.05_[1,28]_=_4.196). Hence, we pooled datasets of these two categories of native breed animals to arrive at a representative site-specific F_m_ value for the region. The GM value for the combined dataset was evaluated to be 3.2 × 10^–3^ d L^-1^. This was similar to the value derived for the dairy farm cattle. Hence, it can be considered the representative value for tropical climatic regions of the Indian subcontinent for radiation dose assessments.

### Comparison of F_m_ values of Sr

A comparison of the F_m_ values derived from this study with those listed in the IAEA^10^ and Howard^12^ is presented in Table 6. The majority of the reported values were derived from the high milk yielding dairy farm cows fed with a significantly higher quantity of nutrient-rich supplement feed. The range given in the IAEA ^10^ from the worldwide published data is 3.4 × 10^–4^—4.3 × 10^–3^ with a GM of 1.3 × 10^–3^ d L^-1^ (GSD = 1.7). These were updated to 1.5 × 10^–5^—4.3 × 10^–3^ with a GM of 1.3 × 10^–3^ d L^-1^ (GSD = 2.1) in Howard^12^ and the IAEA document^52^. Since these two publications have reviewed all the published data in this field, we have listed only these two in Table 6 for comparison. This comparison shows that the data obtained from the present study (3.2 × 10^–3^ d L^-1^) is within the data range presented in the IAEA^10,52^, and the mean is of the same order.

**Table 6:** Comparison of the F_m_ values for Sr.

Region	F_m_ (d L^-1^)		DMI (kg d^-1^, dry mass)	Reference
	Range	Mean (GM)		
Kaiga, India (cows, local breed, raised for this study)	7.5 × 10^–4^-1.4 × 10^–2^	4.7 × 10^–3^ (3.6 × 10^–3^)	9.3 (measured)	Present study
Kaiga, India (cows raised by villagers, local breed)	4.8 × 10^–4^-9.1 × 10^–3^	3.3 × 10^–3^ (2.6 × 10^–3^)	8.3 (measured)	Present study
Kaiga, India (dairy farm, breed cows)	1.8 × 10^–3^—1.1 × 10^–2^	5.5 × 10^–3^ (4.2 × 10^–3^)	13 (measured)	Present study
Representative value for the Indian subcontinent	–	3.2 × 10^–3^	8.3	Recommended from the present study
Worldwide	3.4 × 10^–4^-4.3 × 10^–3^	(1.3 × 10^–3^)	16.1	^10^
Worldwide	1.5 × 10^–5^—4.3 × 10^–3^	1.5 × 10^–3^ (1.3 × 10^–3^)	16.1	^52^
Worldwide (based on IAEA MODARIA, 2016)	1.5 × 10^–5^-4.3 × 10^–3^	1.5 × 10^–3^ (1.3 × 10^–3^)	24.5*	^12^

* Corresponding to the animal of live-weight of 700 kg.

In a preceding publication^35^, we have reported that F_m_ values of stable and radioactive isotopes of Cs (stable Cs and ^137^Cs) for native breed cattle of the Kaiga region were greater by an order of magnitude in comparison with the dairy farm cattle and those published by other investigators, including the value compiled in the IAEA^10^. Possible reasons identified were (i) inadvertent soil ingestion during grazing in the fields as dietary requirements of the cow are met primarily through grazing, and (ii) very low milk yield^35^. However, for Sr, soil ingestion makes no difference to the F_m_ since the F_v_ for grass is greater than unity. In contrast, the F_v_ for Cs isotopes is about 100 times lower. Therefore, soil ingestion will not significantly affect the F_m_ of Sr, unlike Cs^75,84,85^. This is distinctly reflected in the present study; the F_m_ for Sr had the same order of magnitude in all the three categories of cattle studied.

### Concentration ratios (CR)

An alternative method for quantifying grass-to-milk transfer is the concentration ratio (CR); expressed in kg L^-1^ and defined as^10^:

6 $${\text{CR }} = \frac{{{\text{Equilibrium Sr concentration in milk }}\left( {{\text{mg L}}^{{ - {1}}} ,{\text{ fresh mass}}} \right)}}{{{\text{Equilibrium Sr concentration in feed }}\left( {{\text{mg kg}}^{{ - {1}}} ,{\text{ dry mass}}} \right)}}$$

The use of CR for field studies has an advantage since the information on DMI is not essential ^10^, unlike F_m_ calculation. The DMI depends on the size and age of animals^10,12,86^; hence, the F_m_ value is expected to vary across the species and individual animals. On the other hand, the variation of CR between and across the species is minimal.

The CR values thus calculated for Sr (Eq. 6) in this study are compared with the data listed in the IAEA^10,52^ in Table 7. As expected, the CR values were similar among all the categories of cows studied. This comparison of the results also shows that the values observed in the present study are similar to those listed in the IAEA^10,52^.

**Table 7:** CR values for Sr.

Region and details of cows	CR (kg L^-1^)	Reference
Kaiga, India: Cow 1, native breed	1.8 × 10^–2^	Present study
Kaiga India: Cow 2, native breed	2.6 × 10^–2^	Present study
Kaiga, India: raised by the villagers, native breed	2.3 × 10^–2^	Present study
Kaiga India: dairy farm (Holstein Friesian)	5.4 × 10^–2^	Present study
Worldwide, dairy farm	2.3 × 10^–2^	^10^
	2.1 × 10^–2^ (1.7 × 10^–2^) * 5.6 × 10^–4^—1.4 × 10^–1 #^	^12,52^

*Value in the parenthesis is GM value, and others are arithmetic mean.

^#^Represents the range.

Finally, it is important to comment that caution should be exercised on the use of the F_v_ and F_m_ values determined in this study, for environmental equilibrium conditions, to predict the transfer of radioactive Sr isotopes to the food chain during the initial period of a hypothetical emergency situation involving short term excessive release of radioactivity from the nuclear facility. Previous studies^1,87^ at Chernobyl have shown that in the vicinity of the NPP, particularly in the area within the 30 km radius zone, the majority of the fallout from the accident was in the form of particles derived from the uranium dioxide fuel (referred to as ‘hot particles’ or ‘fuel particles’) with low solubility. It was also reported that more than 90% of the release of ^90^Sr was in the form of particles with an average diameter of ~ 10 µm^88^. The predominant mechanism of contamination of grass in such a scenario is dry and wet depositions from the atmosphere. Hence, site-specific studies aimed at establishing a database on interception of dry and wet deposition by grass are essential. Moreover, the migration of ^90^Sr deposited on soil in particle form would differ from naturally present stable Sr under equilibrium conditions^1^ and therefore developing a database on this aspect for tropical regions is also crucial.

## Conclusions

This comprehensive study has established an important database on the F_v_ and F_m_ of stable Sr for field conditions for a tropical and high rainfall region. Most of the data compiled in the IAEA^10,38,52^ are for temperate regions and high milk yielding dairy cows. This study has established the F_m_ values for a cow breed specific to the villages of the Indian subcontinent, which have attributes such as low body mass, very low milk yield, and being fed minimal or no supplement feed. The F_v_ values of stable Sr were similar among two grass species *Pennisetum purpureum* (Schum.) and *Ischaemum indicum* (Houtt.). The site-specific representative value of F_v_ derived from this study was 1.80. The mean value of F_m_ (3.2 × 10^–3^ d L^-1^) derived from this study is within the data range presented in the IAEA document^52^, and the mean is of the same order. The CR values were similar for all the categories of cows studies in this work and those listed in the IAEA documents^10,52^.
